# Unveiling the Hidden Diversity of *Termitomyces* (*Lyophyllaceae*, *Agaricales*) in Northern Thailand: Identification of Five New Species and the First Report of *Termitomyces acriumbonatus*

**DOI:** 10.3390/jof11120830

**Published:** 2025-11-24

**Authors:** Soumitra Paloi, Jaturong Kumla, Wiphawanee Phonrob, Barsha Pratiher Paloi, Nakarin Suwannarach

**Affiliations:** 1 Center of Excellence in Microbial Diversity and Sustainable Utilization, Chiang Mai University, Chiang Mai 50200, Thailand; soumitrabotany@gmail.com (S.P.); jaturong_yai@hotmail.com (J.K.); barshamicrobio@gmail.com (B.P.P.); 2 Myco-Food Laboratory, Food Technology & Science Institute, TCG Centres of Research and Education in Science and Technology, Application Research & Development Center, 54/A/1, Block-DN, Sector-V, Salt Lake City, Kolkata 700091, India; 3 Department of Biology, Faculty of Science, Chiang Mai University, Chiang Mai 50200, Thailand; phwiphawanee@gmail.com; 4 Office of Research Administration, Chiang Mai University, Chiang Mai 50200, Thailand

**Keywords:** Asia, edible mushrooms, new taxa, taxonomy, termite mushrooms, tropical area

## Abstract

Members of the genus *Termitomyces* frequently grow in association with termites. During the monsoon season of 2022 and 2023, a total number of 13 *Termitomyces* samples were collected from the Chiang Mai University campus, Thailand. The objective of this study was to identify the collected samples. Based on morphological and molecular investigations, six distinct species were identified from the collected specimens. Five species (*T. griseobulbus*, *T. griseobrunneus*, *T. planiperforatorius*, *T. pseudoheimii*, and *T. salmonicolor*) are described herein as new to science, while one species (*T. acriumbonatus*) represents a new record for Thailand. The multi-gene phylogenetic analysis of the large subunit (nrLSU) of nuclear ribosomal DNA, the small subunit of mitochondrial DNA (mtSSU), and the nuclear internal transcribed spacer (nrITS) sequences confirmed that all six species belong to the genus *Termitomyces*. Full morphological descriptions, colour photographs, illustrations, and comparisons with phylogenetically and morphologically related species are provided.

## 1. Introduction

Fungi rank second among the major kingdoms in terms of known taxa, with approximately 155,000 species scientifically documented to date [1]; and approximately 2200 edible mushrooms have been reported throughout the world [2]. Among the wild edible mushrooms, most members of the genus *Termitomyces* R. Heim are well-known in different countries around the world, especially in Asia and Africa, due to their distinctive flavours. For example, in China, *Termitomyces* mushrooms are known as “Jizong”, meaning “chicken mushrooms”, and are commonly used as a food source [3]. Regarding their nutritional value, *T. microcarpus* contains a high amount of protein (30.69% of dry weight), fibre (11.60% of dry weight), and various minerals [4]. Medicinally, *T. microcarpus* has shown potential activity against different cancer cell lines [5]. Ethnomedicinally, *T. clypeatus* is used for the treatment of yellow fever in Nepal, pox in India, and constipation and gastritis in Ethiopia [6,7,8].

*Termitomyces* belongs to the family *Lyophyllaceae* Jülich, under the order *Agaricales* Underw. During the middle of the twentieth century, R. Heim established this genus, with *T. striatus* (Beeli) R. Heim as the type species [9]. According to Index Fungorum, there are 55 valid species reported to date [10]. Most of the taxa from *Termitomyces* are reported and described from different countries in Asia and Africa [11]. The highest diversity of *Termitomyces* is found in Africa [12]. Interestingly, *Termitomyces* grows in association with termites, mostly those in the genera *Odontotermes*, *Macrotermes*, and *Microtermes* under the only subfamily *Macrotermitinae* [13]. A diverse range of these termites is frequently found in tropical ecosystems [14]. Recently, many new *Termitomyces* species have been reported from different Asian countries based on morphological characteristics and single- or multi-gene phylogenetic analyses: *T. floccosus* S.M. Tang, Raspé & S.H. Li from Thailand [15]; *T. fragilis* L. Ye, Karun, J.C. Xu, K.D. Hyde & Mortimer, *T. tigrinus* S.M. Tang & Raspé, *T. upsilocystidiatus* S.M. Tang, Raspé & K.D. Hyde, and *T. yunnanensis* S.M. Tang & Raspé from China [3,15,16]; *T. acriumbonatus* Usman & Khalid, *T. sheikhupurensis* Izhar, Khalid & H. Bashir, *T. islamabadensis* S. Ashraf, Usman & Khalid, and *T. pakistanensis* Razaq from Pakistan [17,18,19,20]; *T. gilvus* C.S. Yee & J.S. Seelan from Malaysia [21]; *T. srilankensis* Ediriweera, Voto, Karun. & Kularathne from Sri Lanka [22]; *T. assamicus* L.R. Das, Narzary & A.K. Dutta from India [23]; and *T. dhofarensis* S. Hussain, Al-Yahya’ei, Al-Owaisi & Al-Sadi from Oman [24]. In Thailand, “Hed Khone” is the common name for *Termitomyces*. Different species are collected from local regions and sold in local or street markets, and 14 *Termitomyces* species have been reported from Thailand, including *T. aurantiacus* (R. Heim) R. Heim; *T. bulborhizus* T.Z. Wei, Y.J. Yao, Bo Wang and Pegler; *T. clypeatus* R. Heim; *T. entolomoides* R. Heim; *T. eurrhizus* (Berk.) R. Heim; *T. flavus* S.M. Tang and S.H. Li; *T. floccosus*; *T. fuliginosus* R. Heim; *T. globulus* R. Heim and Gooss.-Font.; *T. heimii* Natarajan; *T. microcarpus* (Berk. and Broome) R. Heim; *T. perforans* R. Heim; *T. radicatus* Natarajan; and *T. striatus* (Beeli) R. Heim [25,26,27,28,29].

Thailand is a biodiversity-rich country in Southeast Asia [30]. Geographically, the northern part is characterised by several mountain ranges and is home to several natural forests covered by a wide range of tree species [31]. Moreover, a total of 36 termite species belonging to 19 genera have been reported from northern Thailand, among which seven *Odontotermes*, four *Macrotermes*, and one *Microtermes* species are known to be associated with *Termitomyces* [32]. Therefore, given the diversity of termite hosts, a correspondingly high diversity of *Termitomyces* species can be expected within these habitats. However, knowledge of *Termitomyces* diversity in northern Thailand remains limited, highlighting the need for additional research. During a survey of macrofungi conducted on the Chiang Mai University campus, Chiang Mai Province, northern Thailand, in 2022 and 2023, 13 specimens of *Termitomyces* were collected. The objective of this study is to characterize and accurately identify these specimens. Based on their morphological characteristics and multi-gene phylogenetic analyses, the specimens were identified as five distinct new species, along with one previously known species.

## 2. Materials and Methods

### 2.1. Sample Collection and Morphological Study

Fresh basidiomata of *Termitomyces* were collected from the Chiang Mai University campus, Chiang Mai Province, northern Thailand, during the rainy seasons (May to August) of 2022 and 2023. The campus covers an area of approximately 229.5 acres and is situated at the foothills of Doi Suthep-Pui National Park. It is characterised by a diverse landscape comprising mixed deciduous and evergreen forests, landscaped gardens, and open grasslands. In the rainy season of 2022, the average daily temperatures ranged from 25 °C to 27 °C, while the relative humidity levels ranged from 79% to 86%. During the rainy season of 2023, the average daily temperature increased to 27 °C to 32 °C, with the relative humidity ranging from 75% to 85%. This weather information was sourced from the Northern Meteorological Center (https://cmmet.tmd.go.th; accessed on 15 April 2025). Macromorphological characteristics, ecological data, and chemical reactions (10% KOH) were noted in the field. Colour codes and terms of Kornerup and Wanscher [33] were used. Specimens were dried at 45 °C for 48 h, depending on the material. Microscopic characteristics were obtained from dried specimens using freehand sections by mounting them in 5% KOH, Congo red, and Melzer’s reagent. They were then viewed through an Olympus CH30 microscope. The size of the basidiospores was documented based on 30 measurements recorded from each specimen. The Q value denotes the length/width ratio of the basidiospores. Basidiospore and Q value statistics are presented as mean values (underlined). The terminology of the microscopic features followed that of Mossebo et al. [34]. The holotype and other examined specimens were deposited at the Chiang Mai University Herbarium of the Department of Biology (CMUB) and the Sustainable Development of Biological Resources Laboratory (SDBR), Faculty of Science, Chiang Mai University, Thailand.

### 2.2. DNA Extraction, PCR Amplification, and Sequencing

Genomic DNA was extracted from fresh specimens using a DNA Extraction Mini Kit (FAVORGEN, Pingtung City, Taiwan), according to the manufacturer’s protocol. The nrITS, nrLSU, and mtSSU regions were amplified by polymerase chain reaction (PCR) using the primer pairs ITS4/ITS5 [35], LROR/LR5 [36], and the *Termitomyces*-specific primers SSUFW105/SSUREV475 [13], respectively. These three domains were amplified in separate PCRs. The PCR programs for nrITS and nrLSU were established by following the methods employed by Paloi et al. [37]. The amplification program for mtSSU consisted of an initial denaturation step at 94 °C for 2 min, followed by 35 cycles of denaturation at 94 °C for 45 s, an annealing step at 59 °C for 45 s, an elongation step at 72 °C for 1 min, and an extension step at 72 °C for 2 min on a peqSTAR thermal cycler (PEQLAB Ltd., Fareham, UK). The PCR products were checked on 1% agarose gels stained with ethidium bromide under UV light. The PCR products were purified using a PCR clean-up Gel Extraction NucleoSpin^®^ Gel and PCR Clean-up Kit (Macherey-Nagel, Düren, Germany), following the manufacturer’s protocol. The purified PCR products were directly sequenced using a genetic analyser at the 1st Base Company (Kembangan, Malaysia) with the PCR primers mentioned above.

### 2.3. Sequence Alignment and Phylogenetic Analyses

Sequences with a high degree of similarity to the newly generated sequences were retrieved from the GenBank database and recent publications [3,13,15,16,17,18,19,20,21,22,23,24,34,38,39,40,41,42], which are detailed in Table 1. Two *Asterophora* and *Lyophyllum* species, *Asterophora lycoperdoides* (Bull.) Ditmar, *Asterophora parasitica* (Bull. ex Pers.) Singer, *Lyophyllum decastes* (Fr.) Singer, and *Lyophyllum shimeji* (Kawam.) Hongo were used for rooting purposes. Multiple sequence alignment was performed using MUSCLE [43] and the default settings. The finalised alignment of the concatenated nrLSU, mtSSU, and nrITS sequences was deposited in Zenodo (https://zenodo.org; accessed on 15 October 2025) under the DOI 10.5281/zenodo.15528282. Phylogenetic analyses were carried out based on the combined dataset of the nrLSU, mtSSU, and nrITS sequences. A phylogenetic tree was constructed using the maximum likelihood (ML) and Bayesian inference (BI) methods. The ML analysis was carried out on RAxML-HPC2 version 8.2.10 [44] on the CIPRES web portal using the GTRCAT model and 1000 bootstrap replications. The BI analysis was performed using MrBayes version 3.2.6 [45]. The best substitution models for the BI analyses were estimated using Akaike Information Criterion (AIC) in jModeltest 2.1.10 [46]. The best substitution models were GTR + G for nrLSU, HKY + I + G for mtSSU, and HKY + G for nrITS. For the BI analysis, six simultaneous Markov chains were run for three million generations with random initial trees, and every 1000 generations were sampled. The burn-in was set to discard the first 2000 trees, and the remaining trees were used to construct the 50% majority-rule consensus phylogram with the calculated Bayesian posterior probabilities (PPs). Branches with bootstrap support (BS) and PP values greater than or equal to 70% and 0.75, respectively, were considered significantly supported [47,48]. Tree topologies were then visualised in FigTree version 1.4.0 [49].

## 3. Results and Discussion

### 3.1. Phylogenetic Analysis

**Figure 1:**
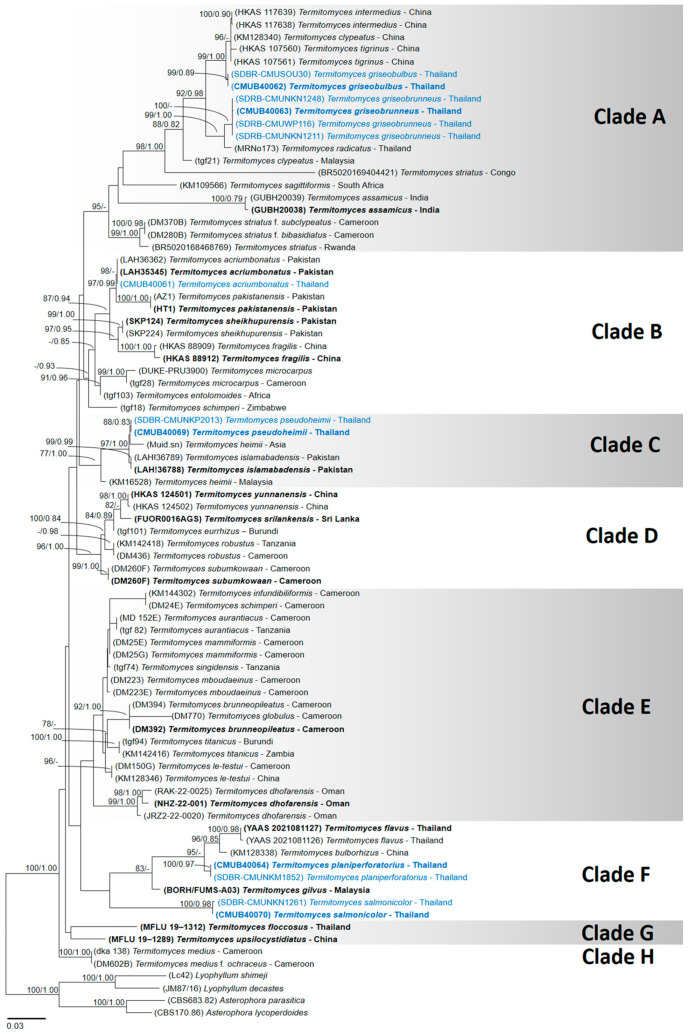
Phylogenetic tree derived from maximum likelihood analysis of 82 taxa of combined nrLSU + mtSSU + nrITS data. *Asterophora lycoperdoides*, *Asterophora parasitica*, *Lyophyllum decastes*, and *Lyophyllum shimeji* were used for rooting purposes. The numbers above branches represent maximum likelihood bootstrap percentages (**right**) and Bayesian posterior probabilities (**left**). Bootstrap values ≥ 70% and Bayesian posterior probabilities ≥ 0.75 are shown above the branches. The scale bar represents the expected number of nucleotide substitutions per site. Type species are highlighted in bold font. Newly reported species in this study are highlighted (blue font).

The newly generated nrITS, nrLSU, and mtSSU sequences were deposited in the GenBank database with the accession numbers shown in Table 1. The combined sequence dataset (nrLSU + mtSSU + nrITS) consisted of a total of 42 species and three varieties of *Termitomyces*, and the aligned dataset comprised 1708 characters including gaps (nrLSU: 1–647; mtSSU: 648–1007; and nrITS: 1008–1708). The best-scoring RAxML tree was established with a final ML value of −9361.118837. The final average standard deviation value of the split frequencies at the end of the total MCMC generations was calculated to be 0.00984 through the BI analysis. The phylograms obtained from the ML and BI analyses exhibited similar topologies. Therefore, the phylogenetic tree obtained from the ML analysis was selected and is presented in this study (Figure 1).

The phylogenetic tree was divided into eight clades (Clade A to H). Clade A represents the closely related species of *T. intermedius* Har. Takah. & Taneyama. The species grouped in this clade are a mix of Asian and African representatives. Morphologically, all species produce medium-sized basidiomata. Our two specimens (CMUB40062 and SDBR-CMUSOU30), introduced as *T. griseobulbus*, formed a monophyletic group with strong support (BS = 99% and PP = 0.89) and are sister to *T. intermedius*, *T. tigrinus*, and *T. radicatus*. However, all these species are morphologically different from *T. griseobulbus*. Another four specimens (CMUB40063, SDBR-CMUNKN1211, SDBR-CMUNKN1248, and SDBR-CMUWP116), introduced as *T. griseobrunneus*, formed a monophyletic group. *Termitomyces griseobrunneus* formed a sister taxon to *T. radicatus* (voucher MRNo173), with sequences deposited based on the Thai collection. However, *T. griseobrunneus* can be easily distinguished from *T. radicatus* by having hymenial cystidia. According to IndexFungorum, the current name of *T. striatus* f. *bibasidiatus* Mossebo is *T. striatus*, but *T. striatus* f. *subclypeatus* Mossebo & Essouman is invalid according to Art. 40.7 of the Melbourne code. Another newly proposed species, *T. assamicus* from India, also clustered into this clade.

Clade B represents *Termitomyces* species that are mostly characterised by a smaller pileus size, and most of the species were described from Asia and a few are from Africa. Our specimen CMUB40061 was placed in *T. acriumbonatus*, described from Pakistan. Morphologically, it was similar to *T. acriumbonatus*. Other well-known *Termitomyces* species belonging to this clade include *T. pakistanensis* Razaq and *T. sheikhupurensis* from Pakistan; *T. fragilis* from China; *T. microcarpus* (Berk. & Broome) R. Heim; and *T. entolomoides* R. Heim from Africa. One sequence of *T. schimperi* (Pat.) R. Heim (voucher tgf18) clustered in this clade, possibly due to misidentified species.

Clade C represents the *T. heimii* complex with high support values (BS = 77% and PP = 1.00) in the phylogenetic tree (Figure 1). All species are medium-sized and were found in the Asian region. Here, two specimens of *T. heimii* (voucher Muid.sn and KM16528) were placed separately in this clade. It is important to recognise the sequence as the type sequence of *T. heimii*, and further detailed study is needed to resolve this complex. It was found that two specimens in this study (CMUB40069 and SDBR-CMUNKP2013), introduced as *T. pseudoheimii*, were monophyletic and formed a sister taxon to *T. heimii* (voucher Muid.sn). Additionally, *T. islamabadensis*, described from Pakistan, was placed in this clade. In Clade D, it included Asian and African *Termitomyces* species. Asian *Termitomyces* species in this clade were recently described, namely *T. srilankensis* and *T. yunnanensis*. Other African *Termitomyces* species belonging to this clade were *T. eurrhizus*; *T. robustus* (Beeli) R. Heim; and *T. subumkowaan* Mossebo. Clade E primarily represents *Termitomyces* species collected from Africa, but Asian specimens of *T. le-testui* and *T. dhofarensis* also belong to this clade.

All sequences of different *Termitomyces* species in Clade F are from the Asian continent. Two specimens (CMUB40064 and SDBR-CMUNKM1852), introduced as *T. planiperforatorius*, formed a monophyletic group with strong support (BS = 100% and PP = 0.97) and are sister to *T. bulborhizus* and unpublished *Termitomyces* sp. (vouchers YAAS 2021081126 and YAAS 2021081127) collected in Thailand. Moreover, two specimens, CMUB40006 and SDBR-CMUNK1760, identified as *T. salmonicolor*, were placed in this clade and formed a monophyletic group, clearly separating them from other species. Two other clades (Clades G and H) represented a few Asian and African *Termitomyces* species, respectively. Two species, *T. floccosus* and *T. upsilocystidiatus*, belonged to Clade G. Another species, *T. medius* R. Heim & Grassé (according to Index Fungorum, *T. medius* f. *ochraceus* Mossebo & Essouman is currently known as *T. medius*), belonged to Clade H.

### 3.2. Taxonomy

#### 3.2.1. *Termitomyces acriumbonatus* Usman & Khalid, Phytotaxa 477: 221 (2020)

**Figure 2:**
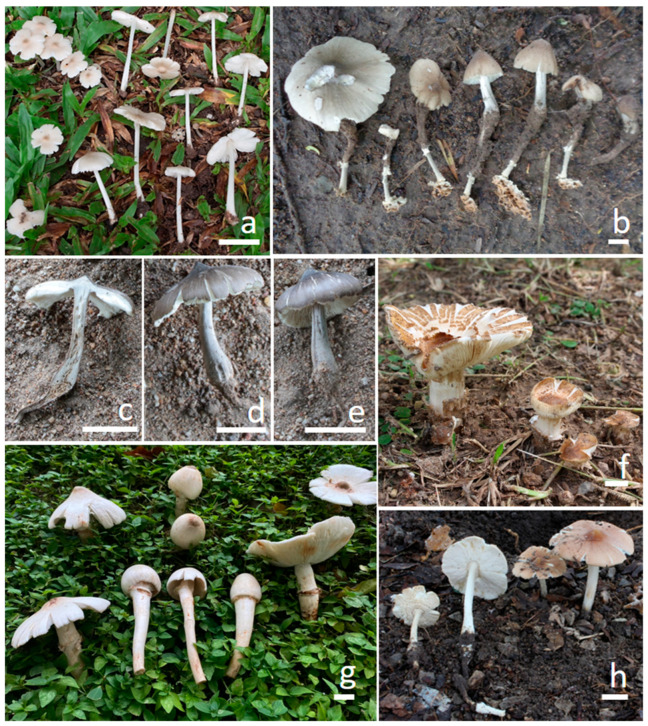
Field photograph of basidiomata. (**a**) *Termitomyces acriumbonatus* (CMUB40061, new record). (**b**) *T. griseobulbus* (CMUB40062, holotype). (**c**–**e**) *T. griseobrunneus* (CMUB40063, holotype). (**f**) *T. planiperforatorius* (CMUB40064, holotype). (**g**) *T. pseudoheimii* (CMUB40069, holotype). (**h**) *T. salmonicolor* (CMUB40070, holotype). Scale bars = 20 mm.

**Figure 3:**
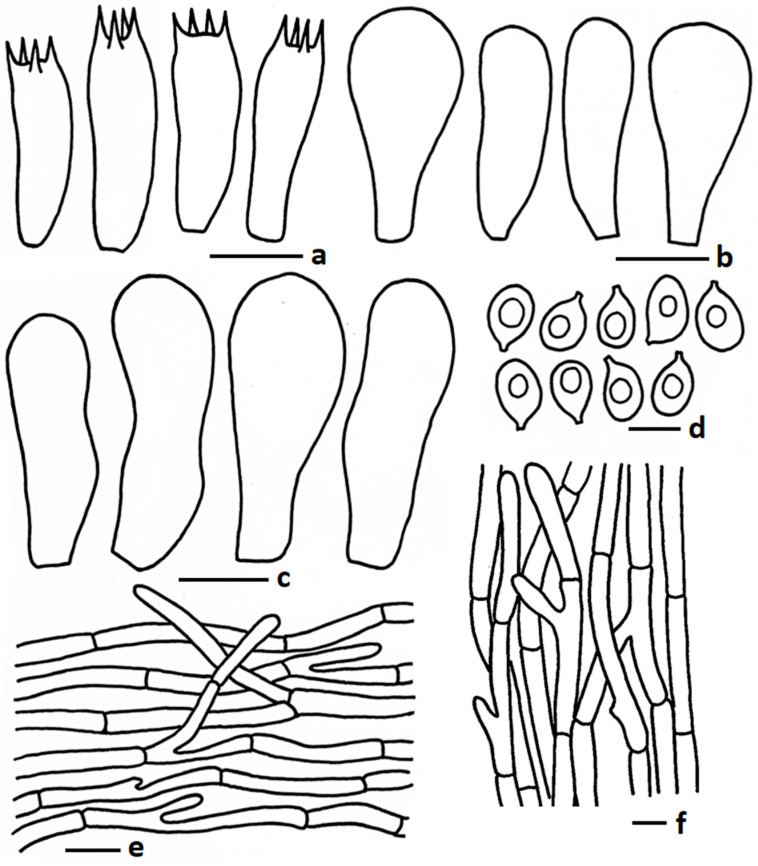
Microscopic features of *Termitomyces acriumbonatus* (CMUB40061, new record): (**a**) basidia; (**b**) cheilocystidia; (**c**) pleurocystidia; (**d**) basidiospores; (**e**) pileipellis; (**f**) stipitipellis. Scale bars: (**a**–**c**,**e**,**f**) = 10 μm and (**d**) = 5 μm.

MycoBank: MB834937 (Figure 2a and Figure 3)

Description: *Pileus* 8–25 mm diam., paraboloid to hemispherical at early stage, becoming broadly convex to applanate to planoconcave; perforatorium prominent, sometimes pointed at early stage, abrupt at maturity; surface smooth at early stage, sometimes smooth or slowly becoming cracked at maturity; colour brownish grey (5D2, 6C2, 7D2) to greyish brown (5D3, 6D2) at centre, fading toward margin orange grey (5B2) to grey (6B1, 7C1) or reddish grey (9B2); margin split at maturity; context thin, white (1A1), no change after exposure to air. *Lamellae* free, 2–3 mm broad, regular, white (1A1) to off white or light grey; edge eroded, concolorous; lamellulae none. *Stipe* 17–55 × 2–3 mm, central, sometimes curved at centre, slightly bulbous at base, smooth, colour white (1A1) to grey (5B1); context white, fibrillose, no change after exposure to air. *Pseudorhiza* more than 20 mm long and 2–3 mm wide, white, extending deeply into and firmly attached to the termite nest. *Odour* mushroom-like.

*Basidiospores* 5.30–6.67–7.86 × 4.02–4.55–5.34 µm, Q = 1.20–1.47–1.63, broadly ellipsoid to ellipsoid, thin-walled hyaline, sometimes 1–2-guttulate oil granules present when viewed with KOH; apiculus short. *Basidia* 16.09–25.50 × 5.50–8.10 µm, clavate to subclavate to sometimes cylindrical, thin walled, hyaline, oil granules present when viewed with KOH, 2–4-spored; sterigmata up to 4.00 µm long, cylindrical. *Lamellae trama* regular, composed of hyphae, and inflating cells, 4.23–11.49 µm wide, thin walled, hyaline, branched, septate. *Cheilocystidia* 18.32–28.00 × 5.81–13.20 µm, utriform to pyriform, thin walled, hyaline. *Pleurocystidia* 15.65–32.12 × 5.81–17.74 µm, same as cheilocystidia. *Pileipellis* two distinct layers; subpellis composed of inflating cells arranged in a chain, 25–110 × 12–25 µm, thin walled, hyaline, some inflated hyphae present, 7.00–12.5 µm broad, thin walled, branched; suprapellis composed of radially arranged hyphae, 2.97–6.50 µm broad, thin walled, hyaline, oil granules present with KOH, branched, septate. *Stipitipellis* composed of tightly arranged hyphae 2.50–8.00 µm broad, hyaline, thin walled, branched, oil granules present with KOH. *Clamp connections* absent in all tissues.

Habit and habitat: Solitary to gregarious, growing on humus-deposited grass field, above subterranean termite nest.

Edibility: Edible.

Material examined: THAILAND. Chiang Mai Province, Chiang Mai University, Angkeaw Reservoir, 18°48′22″ N 98°57′6″ E, elevation 340 m, 8 July 2022, W. Phonrob (CMUB40061). GenBank accession numbers PQ896627 (nrITS), PQ896626 (nrLSU), and PV020670 (mtSSU).

Notes: *Termitomyces acriumbonatus* was recently reported from Pakistan. Morphologically, this species is characterized a small pileus with a brownish pointed papilla, subglobose to ellipsoid basidiospores, and clamp connections in pileipellis and stipitipellis hyphae [17]. The newly collected Thai specimen is morphologically similar to *T. acriumbonatus* in most characteristics including basidiomata size, shape, colour, basidiospores, hymenial cystidia, and pileipellis cell arrangements, but this present collection from Thailand does not show any clamp connections in the pileipellis or stipitipellis. However, most of the morphological character similarities as well as phylogenetic placement with *T. acriumbonatus* suggest that the present Thai collection is conspecific with *T. acriumbonatus*. The morphologically similar species, *T. microcarpus*, can cause confusion in the field. However, *T. microcarpus* has a smaller pileus (up to 15 mm diam.) and *T. acriumbonatus* have brownish grey to greyish brown perforatorium [9].

*Termitomyces pakistanensis*, *T. sheikhupurensis*, and *T. fragilis* are closely related to *T. acriumbonatus*. *Termitomyces pakistanensis* has a smaller pileus (1.8–2 cm diam.), an umbonate cover with squamulose at the young stage, adnexed lamellae attachment, no pseudorhiza, and a pileus covered with larger terminal elements (35.5–76.5 × 10.5–28.5 μm) [20]. *Termitomyces sheikhupurensis* has a similar basidiomata size but white velar remnants present on the pileus margin, a stipe surface pale yellowish toward the pileus, a short pseudorhiza, and thin suprapellis hyphae (1–3 µm) [17]. *Termitomyces fragilis*, recently reported from China, has a fragile stipe, very long pseudorhiza (up to 72 mm), larger basidiospores (9.0–10.5 × 5.5–7.5 µm), and larger hymenial cystidia (40–80 × 12–31 µm) [3].

#### 3.2.2. *Termitomyces griseobulbus* Paloi & N. Suwannar., sp. nov.

**Figure 4:**
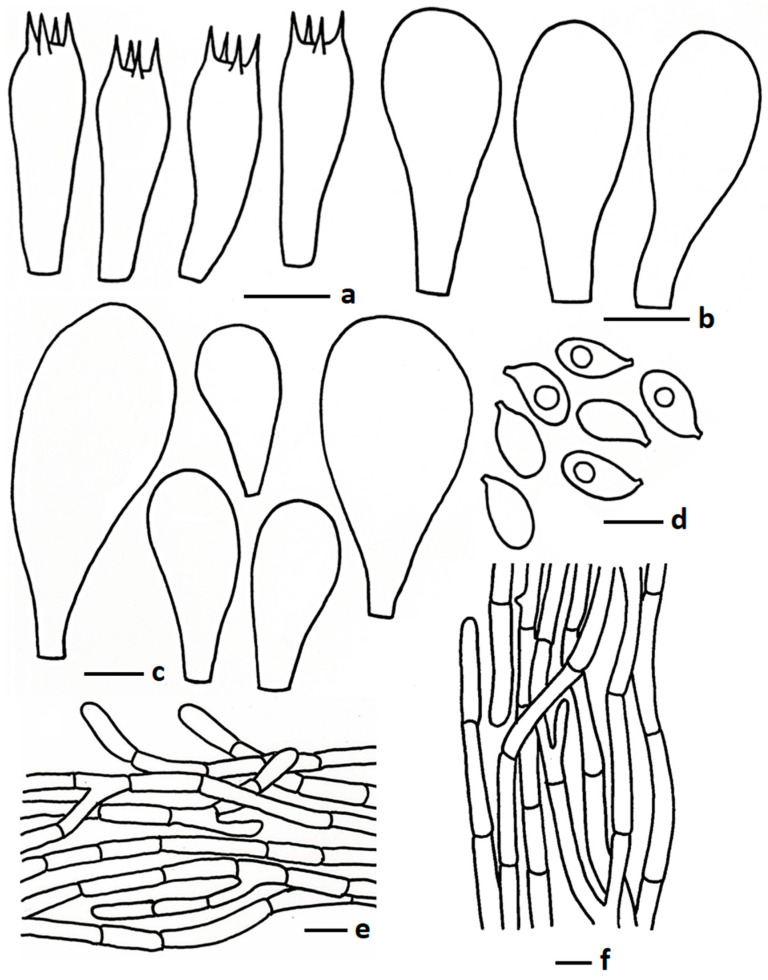
Microscopic features of *Termitomyces griseobulbus* (CMUB40062, holotype): (**a**) basidia; (**b**) cheilocystidia; (**c**) pleurocystidia; (**d**) basidiospores; (**e**) pileipellis; (**f**) stipitipellis. Scale bars: (**a**–**c**,**e**,**f**) = 10 μm and (**d**) = 5 μm.

MycoBank: MB857473 (Figure 2b and Figure 4)

Holotype: THAILAND. Chiang Mai Province, Chiang Mai University, opposite to the volleyball court, 18°47′58.7″ N 98°57′25.2″ E, elevation 330 m, 16 August 2022, S. Paloi (CMUB40062). GenBank accession numbers PQ900075 (nrITS), PQ896628 (nrLSU), and PV020671 (mtSSU).

Diagnosis: Differs from closely related species by having a medium, brownish grey to grey pileus, free lamellae attachment, basidiospores 5.00–8.10 × 3.55–4.77 µm, basidia 16.00–27.50 × 5.90–9.44 µm, hymenial cystidia, and pileipellis composed of suberect to repent hyphae.

Etymology: “*griseobulbus*” represents the grey-coloured bulb present at the stipe base.

Description: *Pileus* 46–80 mm diam., obtusely conical, umbonate to hemispherical or sometimes campanulate at young stage, becoming convex, sub umbonate to applanate or plano concave with a central umbo at maturity; perforatorium round or sometimes blunt pointed; surface moist, velutinous to pubescent; colour brownish grey (5D2) to greyish brown (5D3) at very early stage, brownish grey (5D2, 7E2) to greyish brown (5D3) or grey (7D1) at centre becoming faded toward margin pale grey (1B2), medium grey (1E1), grey (2B1) to reddish grey (7B1) or sometimes similar to centre, no change after bruising; margin wavy at maturity, cracked, up to ½ of pileus, straight to inflexed; context up to 3 mm thick at middle, white to cream, no change after exposure to air. *Lamellae* free, up to 3 mm broad, densely crowded, regular, white (1A1) at young stage becoming, pale grey (1A2) to yellowish grey (2C2); edge serrate or eroded, concolorous; 2–3 lamellulae. *Stipe* 40–55 × 6–9 mm, central, sometimes slightly curved at centre, twisted at maturity, light grey (1C1) or medium grey (1E1) to grey (2C1), sometimes white (1A1) at early stage. turned light brownish with KOH; surface scabrous, moist, near ground forming a bulbous base, concolorous with stipe, sometimes darker than stipe; context solid, fibrillose, white, no change after exposure to air. *Pseudorhiza* 60–75 × 3–5 mm, white (1A1) or similar to the stipe, surface sometimes pubescent; context solid, white, extending deeply into and firmly attached to the termite nest. *Odour* mushroom-like.

*Basidiospores* 5.00–6.76–8.10 × 3.55–3.99–4.77 µm, Q = 1.39–1.69–1.93, ellipsoid, thin walled, hyaline, oil granules present when viewed with KOH, sometimes 1–2-guttulate. *Basidia* 16.00–27.50 × 5.90–9.44 µm, clavate to subclavate, sometimes subcylindrical, thin walled, hyaline, 4-spored; sterigmata 1.09–2.5 µm long, cylindrical, sometimes obtuse apex. *Lamellae trama* regular, composed of hyphae, 6.50–14.00 µm broad, thin walled, hyaline, branched, septate. *Cheilocystidia* 23.50–39.50 × 8.75–14.5 µm, pyriform to clavate with round apex, frequently present in mature stage, thin walled, hyaline. *Pleurocystidia* two types, big pyriform, 51.50–79.50 × 17.75–32.00 µm, not frequent, thin walled, hyaline; small pyriform, with rounded apex, 30.50–49.00 × 13.50–26.50 µm, thin walled, hyaline, frequently present. *Pileipellis* two distinct layers; subpellis composed of hyphae, 5.50–12.00 µm wide, thin walled, hyaline, branched, hyaline; suprapellis composed of tightly arranged hyphae, 3.50–6.00 µm wide, sub erect to repent, sometimes few erect, thin walled, hyaline, oil granules present with KOH, branched, hyaline, hyphal end obtuse, sometimes round apex. *Stipitipellis* composed of hyphae, 3.50–12.00 µm wide, thin walled, hyaline, septate, branched. *Clamp connections* absent in all tissues.

Habit and habitat: Gregarious, rarely solitary, growing above subterranean termite nests.

Edibility: Edible.

Additional material examined: THAILAND. Chiang Mai province, Chiang Mai University, near CMU football ground, 18°47′54.7″ N 98°57′38.2″ E, elevation 323 m, 20 August 2022, S. Paloi (SDBR-CMUSOU30). GenBank accession numbers PQ900076 (nrITS), PQ896629 (nrLSU), and PV020672 (mtSSU).

Notes: Morphologically and phylogenetically closely related species are *T. intermedius*, *T. tigrinus*, *T. griseobrunneus*, and *T. radicatus*. *Termitomyces intermedius* has a dark grey pileus, larger basidiospores (9.0–14.9 × 5.3–10.2 µm) and basidia (43–68 × 10–20 µm), and larger (40–169 × 19–34 µm) oblong, obovoid, or ellipsoid pleurocystidia [16]; *T. tigrinus* contains adnexed lamellae attachment and an ixocutis type of pileipellis and no pleurocystidia [16]; *T. griseobrunneus* differs by having a smaller pileus (25–45 mm in diam.) with a fibrillose surface, thin pileus context (1 mm), and smaller stipe (in this study). However, *T. radicatus* has a smaller (35 mm in diam.) orange white to orange grey pileus, short pseudorhiza (25 mm long), and no hymenial cystidia [50]. *Termitomyces entolomoides* is a somewhat morphologically similar species but differs by having a smaller pileus (30–40 mm in diam.) and a greyish pseudorhiza [51]. According to Wei et al. [52], *T. entolomoides* has larger cheilocystidia (28–60 × 13.0–31 µm) and rarely pleurocystidia.

#### 3.2.3. *Termitomyces griseobrunneus* Paloi, W. Phonrob & N. Suwannar., sp. nov.

**Figure 5:**
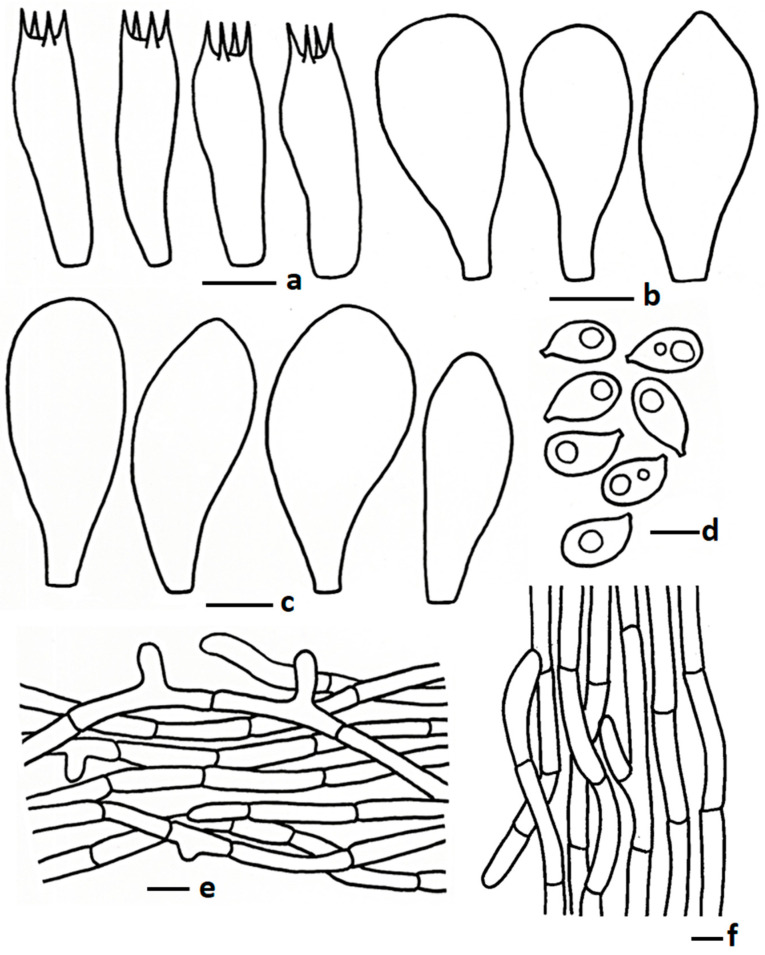
Microscopic features of *Termitomyces griseobrunneus* (CMUB40063, holotype): (**a**) basidia; (**b**) cheilocystidia; (**c**) pleurocystidia; (**d**) basidiospores; (**e**) pileipellis; (**f**) stipitipellis. Scale bars: (**a**–**c**,**e**,**f**) = 10 μm and (**d**) = 5 μm.

MycoBank: MB857474 (Figure 2c–e and Figure 5)

Holotype: THAILAND. Chiang Mai Province, Chiang Mai University campus, Plam Garden, 18°48′1″ N 98°57′19″ E, elevation 350 m, 18 July 2022, W. Phonrob (CMUB40063). GenBank accession numbers PQ899488 (nrITS), PQ896878 (nrLSU), and PV020673 (mtSSU).

Diagnosis: Brownish grey to dark brown pileus with pointed perforatorium, comparatively short stipe (22–35 × 4–9 mm) with bulbous base, basidiospores 5.50–8.25 × 3.50–5.40 µm, and two different types of cheilocystidia.

Etymology: “*griseobrunneus*” indicates a grey-brown coloured pileus.

Description: *Pileus* 25–45 mm diam., campanulate to papillate at young, becoming broadly convex to applanate or plano convex with pointed perforatorium at centre when mature; surface moist, shiny when dry, fibrillose-like, cracked, brownish grey (6E2, 8F2) to grey (8F1), greyish brown (8F3), dark brown (8F4) at centre, becoming faded toward margin grey (3C1) to greyish brown (6D2, 7C1, 11F1), brownish grey (9F2), greenish grey (30B2) or sometimes similar to the centre at early stage, no change after bruising; margin inflexed at young, becoming straight at maturity, wavy, cracked; context 1mm wide at middle, white to off-white, no change after exposure to air. *Lamellae* free, up to 6 mm broad, regular, white (1A1) at early stage, becoming brownish grey (5B2) at mature stage; edge serrate to eroded, concolorous; 2–3 lamellulae. *Stipe* 22–35 × 4–9 mm, central, sometimes curved at centre, tapering upward, base slightly bulbous to sometimes district bulbous, up to 16 mm wide; surface pubescent to minutely ribbed when mature, White (1A1) at early stage to grey (3D1, 7C1), sometimes violet grey (18D2) to greyish violet (19C3) at maturity, no change after bruising; context white to off-white, solid, fibrillose, no change after exposure to air. *Pseudorhiza* 25–55 × 2–5 mm, sometimes similar to the stipe or yellowish grey (3B2) to golden grey (4C2) or grey (4C1), solid, extending deeply into and firmly attached to the termite nest. *Odour* mushroom-like.

*Basidiospores* 5.50–7.10–8.25 × 3.50–4.40–5.40 µm, Q = 1.39–1.64–1.88, ellipsoid, thin walled, hyaline, oil granules present when viewed KOH, 1–2-guttulate, apiculus very short. *Basidia* 19.50–28.00 × 5.50–8.50 µm, clavate to subclavate or sometimes subcylindrical, thin walled, hyaline, oil granules present, 2–4-spored; sterigmata 1.50–3.50 µm long, cylindrical. *Lamellae trama* regular, composed of inflated hyphae, 5.00–13.00 µm wide, thin walled. *Cheilocystidia* 20.50–33.00 × 10.50–15.50 µm, pyriform, frequent; 24.50–37.00 × 8.50–13.50 µm, moniliform to short appendiculate or sometimes broadly clavate, thin walled, hyaline. *Pleurocystidia* 34.50–80.50 × 18.00–41.50 µm, pyriform, frequent; 31.50–43.00 × 11.50–16.00 µm, clavate with obtuse or less pointed apex, thin walled, hyaline. *Pileipellis* two layers; subpellis composed of inflated hyphae and/or chain of inflated cell, 5.00–11.50 µm wide, thin walled, septate; suprapellis up to 100 µm deep, composed of tightly arranged sub-erect or repent hyphae, 3.00–6.50 µm wide, thin walled, hyaline, branched, septate, oil granules present with KOH, hyphal end obtuse to sometimes round. *Stipitipellis* composed of tightly arranged hyphae, 3.00–8.50 µm wide, thin walled, hyaline, branched, septate, hyphal end obtuse. *Clamp connections* absent in all tissues.

Edibility: Edible.

Habit and habitat: Solitary to gregarious, growing on soil above subterranean termite nests.

Additional material examined: THAILAND. Chiang Mai Province, Chiang Mai University, Sala Dhamma, 18°48′15″ N; 98°57′10″ E, elevation 340 m, 19 July 2022, W. Phonrob (SDBR-CMUWP116), GenBank accession numbers PQ896901 (nrLSU), PV020674 (mtSSU); Chiang Mai University, Sala Dhamma, 18°48′15″ N; 98°57′10″ E, elevation 340 m, 25 July 2023 N. Suwannar. (SDBR-CMUNKN1211); GenBank accession numbers PQ899491 (nrLSU) and PV020675 (mtSSU); Chiang Mai University, Sala Dhamma, 18°48′15″ N; 98°57′10″ E, elevation 340 m, 10 August 2023, W. Phonrob (SDBR-CMUNKN1248). GenBank accession numbers PQ899488 (nrITS), PQ896878 (nrLSU), and PV020673 (mtSSU).

Notes: Morphologically and phylogenetically, *T. griseobrunneus* is very close to some Chinese and Thai species, such as *T. intermedius*, *T. tigrinus*, *T. griseobulbus*, and *T. radicatus*. *Termitomyces intermedius* is easily distinguished from these species by having a larger pileus (40–110 mm diam.), a rimose-squamulose surface on the pileus (dry condition), and larger basidiospores (9.0–14.9 × 5.3–10.2 μm) and basidia (43–68 × 10–20 μm), but the average Q value is similar in all these species, and they all have an ixocutis pileipellis [16]; *T. tigrinus* has a larger pileus (70–90 mm diam.), adnexed lamellae attachments, no pleurocystidia, and an ixocutis type of pileipellis [16]. *Termitomyces griseobulbus* differs from *T. griseobrunneus* by having a larger pileus size (46–80 mm in diam.), a velutinous to pubescent pileus surface, a comparatively thicker pileus context (3 mm), and a longer stipe (40–55 mm) with a prominent scabrous surface (in this manuscript). *Termitomyces radicatus*, originally described from India, differs based on characteristics like an orange white to orange grey pileus, yellow white to orange white stipe, and no hymenial cystidia [50]. The bulbous stipe base species *T. bulborhizus* differs by having a larger pileus (50–220 mm in diam.), a broadly round or blunt pointed perforatorium, and a longer pseudorhiza (800 mm) [53]; another species, *T. gilvus*, described from Malaysia, has a larger basidiomata, a thick pileus context (40 mm), no pleurocystidia and clamp connections [21].

#### 3.2.4. *Termitomyces planiperforatorius* Paloi & N. Suwannar., sp. nov.

**Figure 6:**
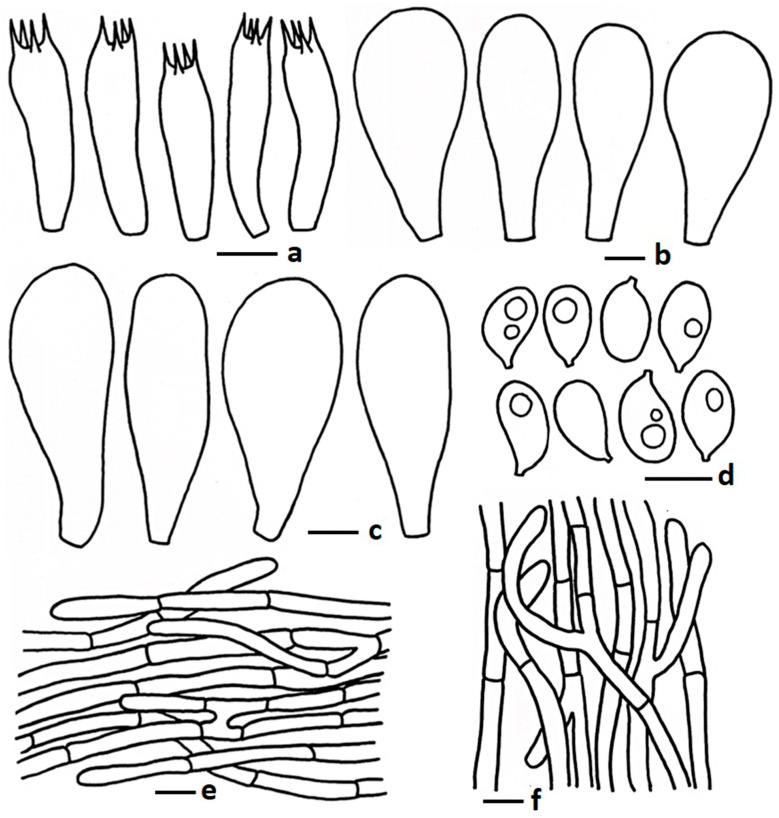
Microscopic features of *Termitomyces planiperforatorius* (CMUB40064, holotype): (**a**) basidia; (**b**) cheilocystidia; (**c**) pleurocystidia; (**d**) basidiospores; (**e**) pileipellis; (**f**) stipitipellis. Scale bars: (**a**–**c**,**e**,**f**) = 10 μm and (**d**) = 5 μm.

MycoBank: MB857475 (Figure 2f and Figure 6)

Holotype: THAILAND. Chiang Mai Province, Chiang Mai University campus, near Female Dormitory 3, 18°47′58.7″ N 98°57′11.4″ E, elevation 341 m, 4 August 2022, S. Paloi (CMUB40064). GenBank accession numbers PQ896983 (nrLSU) and PV020677 (mtSSU).

Diagnosis: Differs from closely related species by having a medium-sized pileus with a slightly round to flat but never pointed perforatorium, a cracked pileus surface when mature and the appearance of squamules on the surface, cheilocystidia 27.12–56.50 × 10.50–29.50 µm, and pyriform to utriform or clavate pleurocystidia with a round apex.

Etymology: “*planiperforatorius*” refers to the flat perforatorium present on the pileus surface.

Description: *Pileus* 22–97 mm diam., convex to broadly convex at early stage, becoming plano concave to concave at maturity; perforatorium slight round to flat, never pointed, moist to dry; surface greyish orange (5B4), brownish orange (5C3), greyish brown (6D3), to light brown (6D4), no change after bruising, smooth to slightly pubescent, cracked, look squamose-like, arranged as zonate, easily pilled; margin split, sometimes revolute to reflexed at maturity; context white (1A1) to cream, up to 2 mm thick at middle, no change after exposure to air. *Lamellae* free, regular, sometimes bifurcate, white (1A1) at early age to greyish white (1B1) at maturity; edge serrate to eroded, concolorous; 2 lamellulae. *Stipe* 55–72 × 7–19 mm, central, sometimes slightly curved at centre, more or less equal, sometimes slightly bulbous at base; surface scabrous, golden grey (4C2) to brownish grey (5D2) or greyish brown (5D3), no change after bruising; context solid, fibrillose, white to cream, no change after exposure to air. *Pseudorhiza* 35–50 × 3–5 mm, similar with stipe colour, sometimes off-white, extending deeply into and firmly attached to the termite nest. *Odour* mild.

*Basidiospores* 5.00–6.40–7.60 × 3.40–3.95–4.50 µm, Q = 1.33–1.63–2.10, broadly ellipsoid to ellipsoid, thin walled, hyaline, oil granules present when viewed with KOH, 1–2-guttulate. *Basidia* 19.50–30.50 × 5.25–7.50 µm, subclavete to subcylindrical, sometimes clavate, thin walled, hyaline, 2–4-spored; sterigmata 1.00–3.00 µm long, pointed apex or sometimes obtuse apex. *Lamellae trama* regular, composed of inflated hyphae, 3.75–12.50 µm wide, thin walled. *Cheilocystidia* 27.12–56.50 × 10.50–29.50 µm, mostly pyriform to sometimes utriform or clavate, thin walled, hyaline. *Pleurocystidia* different types; pyriform, 47.00–69.50 × 17.50–26.00 µm, not very frequent; utriform 39.50–51.50 × 12.00–16.50 µm, frequent; clavate with round apex 41.50–54.50 × 16.00–23.50 µm, rare; thin walled, hyaline. *Pileipellis* two distinct layers; subpellis composed of radially arranged hyphae, thin walled; suprapellis up to 90 mm deep, composed of sub-erect to repent hyphae, 3.00–8.25 µm wide, thin walled, hyaline, branched, septate, hyphal end obtuse. *Stipitipellis* composed of tightly arranged hyphae, 4.0–10.00 µm wide, thin walled, hyaline, branched, septate, hyphal end obtuse or round. *Clamp connections* absent in all tissues.

Edibility: Edible.

Habit and habitat: Solitary to gregarious, growing on grass fields above subterranean termite nests.

Additional material examined: THAILAND. Chiang Mai province, Chiang Mai University, near Tat Chomphu Reservoir, 18°48′16.0″ N 98°57′08.2″ E, elevation 333 m, 11 August 2022, S. Paloi (SDBR-CMUNKM1852). GenBank accession numbers PQ896985 (nrLSU) and PV020678 (mtSSU).

Notes: Morphologically and phylogenetically, it is close to *T. bulborhizus* and *T. gilvus*. *Termitomyces bulborhizus*, originally described from China, differs from the present species by having a larger pileus (100–220 mm in diam.) with reddish brown to dark brown centre, fading from pale brown to brown toward the margin, prominent globose bulbous stipe base, larger cheilocystidia (19–60 × 12–34 µm) [52]; *T. gilvus* differs by having a dark brown, blunt pointed perforatorium on the pileus, a thicker pileus context (40 mm), a prominent globose bulbous base on the stipe, and clamp connections [20]. Some other look-alike species are *T. umkowaan* (Cooke & Massee) D.A. Reid and *T. globulus* R. Heim & Gooss.-Font. *Termitomyces umkowaan*, described from South Africa and widely distributed in Asia and Africa, which differs by having a larger pileus (80–220 mm diam.), a bulbous stipe base, comparatively larger basidiospores (6.1–13 × 4.3–6 µm), and narrow suprapellis hyphae (3.1–4.7 µm wide) [54]. Another species, *T. globulus*, differs by having a larger (150–200 mm in diam.) subglobose to bell-shaped pileus and a larger but not prominent perforatorium [50].

#### 3.2.5. *Termitomyces pseudoheimii* Paloi, N. Suwannar. & J. Kumla sp. nov.

**Figure 7:**
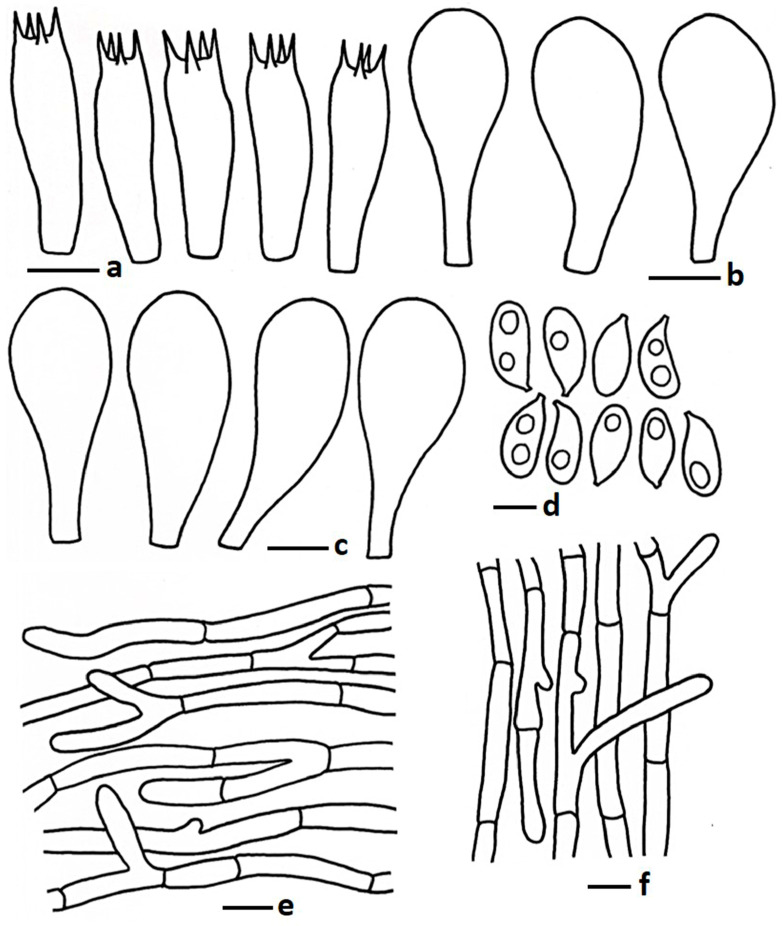
Microscopic features of *Termitomyces pseudoheimii* (CMUB40069, holotype): (**a**) basidia; (**b**) cheilocystidia; (**c**) pleurocystidia; (**d**) basidiospores; (**e**) pileipellis; (**f**) stipitipellis. Scale bars: (**a**–**c**,**e**,**f**) = 10 μm and (**d**) = 5 μm.

MycoBank: MB857476 (Figure 2g and Figure 7)

Holotype: THAILAND. Chiang Mai Province, Chiang Mai University, 18°47′49″ N 98°57′30″ E, elevation 332 m, 20 August 2022, N. Suwannarach (CMUB40069). GenBank accession numbers PQ897224 (nrITS), PQ897223 (nrLSU), and PV020679 (mtSSU).

Diagnosis: Differs from closely related species by having a smooth to velutinous pileus surface, ellipsoid to elongate basidiospores (7.53–10.89 × 3.67–6.13 µm, Q = 1.60–3.6), and comparatively wider suprapellis hyphae 4.22–8.2 µm wide.

Etymology: “*pseudoheimii*”—“*pseudo*” means false, referring to the morphological characteristics that are easily mistaken for *T. heimii*.

Description: *Pileus* 29–99 mm diam., hemispherical to convex at early stage, becoming broadly convex to subumbonate or applanate with apical papilla; perforatorium not pointed, surface smooth to velutinous, moist, brownish grey (5C2), greyish brown (5D4) to light brown (6D4) or brown (6E4) at centre (papilla), white (1A1) to sometimes orange white (5A2) at early stage, becoming white (1A1) at maturity, sometimes brownish tent present; margin cracked up to half of the pileus; context white (1A1), unchanged when exposure to air. *Lamellae* free, up to 5 mm broad, regular, white (1A1), becoming slightly yellowish white (4A2) to orange white (5A2) at maturity, sometimes light brownish after handling; edge smooth to wavy or serrate at maturity, concolorous; 2–3 lamellulae in a series. *Stipe* 45–65 × 7–12 mm, central, sometimes curved, smooth, white (1A1), to very light brown toward base at maturity; context white, fibrillose, no change after exposure to air; annulus present, light brown (6D4) to brown (5E4–F4). *Pseudorhiza* up to 170 mm long and 8–10 mm wide, white (1A1) to off white or brownish grey (5C2) to brownish orange (5C3), extending deeply into and firmly attached to the termite nest. *Odour* mild.

*Basidiospores* 7.53–9.33–10.89 × 3.67–4.67–6.13 µm, Q = 1.60–2.02–2.33, ellipsoid to elongate, thin walled, hyaline, sometimes 1–3-guttules, apiculus short, inamyloid. *Basidia* 15.75–26.5 × 6.91–8.75 µm, clavate to sub–clavate, thin walled, hyaline, oil granules present with KOH, mostly 2–4-spored, rarely 1-spored; sterigmata 1.0–4.5 µm long, cylindrical. *Lamellae trama* regular, composed of hyphae, 6.0–12.5 µm broad, thin walled, hyaline, branched, septate. *Cheilocystidia* 18.5–35.0 × 8.0–12.5 µm, pyriform to sometimes broadly clavate, thin walled, hyaline. *Pleurocystidia* 32.5–41.75 × 11.5–15.25 µm, pyriform, thin walled, hyaline. *Pileipellis* two distinct layers; subpellis composed of radially arranged chain of ellipsoids cells, 6.01–11.0 µm broad, thin walled, hyaline, branched; suprapellis up to 250 µm deep, composed of sub-erect to repent hyphae, 4.22–8.2 µm broad, thin walled, hyaline, branched, septate, hyphal end obtuse, gelatinised. *Stipitipellis* interwoven hyphae, 3.32–8.0 µm broad, thin walled, hyaline, branched, septate, oil granules present with KOH. *Clanp connections* absent in all tissues.

Habit and habitat: Gregarious, growing on grass fields above subterranean termite nests.

Edibility: Edible.

Other material examined: THAILAND. Chiang Mai Province, Chiang Mai University, 18°47′49″ N 98°57′30″ E, elevation 332 m, 13 September 2022, N. Suwannarach (SDBR-CMUNKP2013). GenBank accession numbers PQ897225 (nrITS), PQ897226 (nrLSU), and PV020680 (mtSSU).

Notes: Morphologically, *T. pseudoheimii* is close to *T. heimii* and *T. islamabadensis*. *Termitomyces heimii* is a well-known edible mushroom in several Asian countries and differs from the present species due to velar squamules covering the pileus and stipe surfaces, smaller basidiospores, and pileipellis repent epicutis formed by narrow hyphae (2.0–4.5 µm) [52,55]. Previously, Jannual et al. [27] described *T. heimii* from Thailand as having a comparatively very small pileus (up to 35 mm diam.) with a smaller stipe length (up to 40 mm long), and smaller-diameter pileipellis hyphae (2.0–4.5 µm). Another *T. heimii* from Kanchanaburi Province, Thailand, has smaller basidiospores (5–7 × 3.5–4.5 µm) [26], but *T. pseudoheimii* differs from these described species due to its larger pileus (up to 99 mm in diam.) and basidiospore size (7.53–10.89 × 3.67–6.13 µm). *Termitomyces islamabadensis* differs from the present species due to a creamy white or whitish grey pileus margin, 0 to 2 lamellulae, obovoid to lacrymoid basidiospores (Q value = 1.45–1.8), and comparatively narrow pileipellis hyphae (3.5–4.6 µm broad) [18]. Based on basidiomata size and colour, *T. mammiformis* R. Heim and *T. upsilocystidiatus* can cause confusion with *T. pseudoheimii* in the field. *Termitomyces mammiformis* has unique characteristics: mammiform, scrobiculate perforatorium on the pileus, and pale grey to pale brown velar squamules present on the pileus surface when mature [52,56]. Additionally, *T. upsilocystidiatus*, recently described from China, differs from *T. pseudoheimii* by having smaller basidiospores (4.8–6.8 × 3.5–4.2 μm) and Y-shaped cheilocystidia [15].

#### 3.2.6. *Termitomyces salmonicolor* Paloi, W. Phonrob & N. Suwannar., sp. nov.

**Figure 8:**
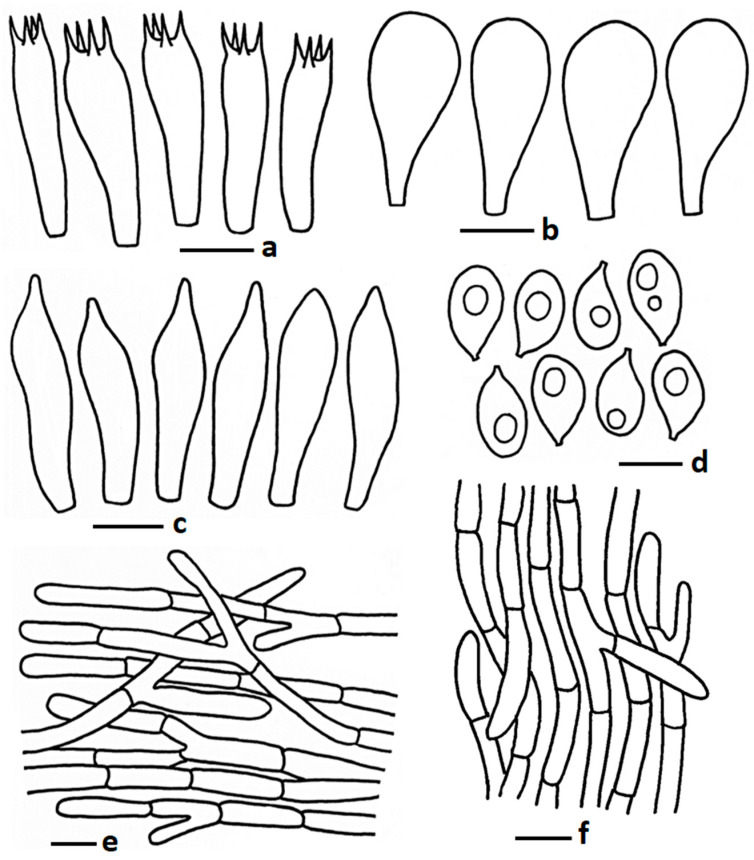
Microscopic features of *Termitomyces salmonicolor* (CMUB40070, holotype): (**a**) basidia; (**b**) cheilocystidia; (**c**) pleurocystidia; (**d**) basidiospores; (**e**) pileipellis; (**f**) stipitipellis. Scale bars: (**a**–**c**,**e**,**f**) = 10 μm and (**d**) = 5 μm.

MycoBank: MB857477 (Figure 2h and Figure 8)

Holotype: THAILAND. Chiang Mai Province, Chiang Mai University, 18°47′45.5″ N 98°57′21.8″ E, elevation 332 m, 4 August 2022, S. Paloi and W. Phonrob (CMUB40070). GenBank accession numbers PQ897227 (nrLSU) and PV020681 (mtSSU).

Diagnosis: Differs from closely related species by having a smaller pileus with a smooth to velutinous surface, a slight to absent bulbous base, smaller basidiospores (4.67–6.68 × 3.10–4.26 µm), cheilocystidia 16.25–29.50 × 8.50–10.50 µm, and without clamp connections.

Etymology: “*salmonicolor*” refers to the light salmon colour of the pileus.

Description: *Pileus* 30–62 mm diam., convex, umbonate to papillate at young stage, becoming broadly convex to applanate with a pointed perforatorium at centre when mature; surface smooth to velutinous, moist, light brown (6D4), brown (6E5), dark brown (7F5) at centre, becoming faded toward margin, orange grey (6B2), brownish grey (6C2), reddish grey (7B2), greyish red (7B3), brownish orange (7C3) sometimes reddish white (8A2) or dull red (8C3); no change after bruising; margin regular, wavy, cracked, sometimes inflexed; context 1 mm thick at middle, white (1A1) to cream, no change after exposure to air. *Lamellae* free, regular, sometimes frocked near margin, white (1A1) at early stage, becoming greyish white (1A2), yellowish grey (4B2) at maturity; edge eroded, concolorous, 1–2 lamellulae. *Stipe* 34–65 × 5–12 mm, central, sometimes slightly curved a centre, base slightly bulbous or sometimes more or less equal, surface smooth to longitudinally striate, white (1A1) and greyish white (1A2) to yellowish grey (4B2) or grey (5B1); no change after bruising; context white to off-white, fibrillose, no change after exposure to air. *Pseudorhiza* 30–45 × 2–4 mm, similar colour as stipe or sometimes orange grey (5B2) to greyish orange (5B3); context solid, extending deeply into and firmly attached to the termite nest. *Odour* mild.

*Basidiospores* 4.67–5.75–6.68 × 3.10–3.68–4.26 µm, Q = 1.33–1.57–1.84, broadly ellipsoid to ellipsoid, thin walled, hyaline, oil granules present when viewed with KOH, 1–2-guttulate, short apiculus. *Basidia* 17.00–31.50 × 5.50–8.00 µm, clavate to sub clavate or sometimes subcylindrical, thin walled, hyaline, oil granules present with KOH, 2–4-spored; sterigmata 1.00–2.75 µm long, cylindrical. *Lamellae trama* regular, composed of inflated hyphae, 5.00–15.50 µm wide, thin walled, hyaline. *Cheilocystidia* 16.25–29.50 × 8.50–10.50 µm, pyriform, sometimes broadly clavate with round apex, thin walled, hyaline. *Pleurocystidia* 23.50–32.50 × 5.75–8.00 µm, short appendiculate to moniliform or pointed apex, thin walled, hyaline. *Pileipellis* two layers; subpellis composed of a chain of inflated cells, 9.60–24.15 µm wide, thin walled, hyaline, septate; suprapellis up to 80 µm deep, composed of erect to sub-erect or repent hyphae, 4.67–8.50 µm wide, thin walled, hyaline, septate, branched, hyphal end obtuse to sometimes round. *Stipitipellis* composed of tightly arranged hyphae, 3.11–6.00 µm wide, thin walled, hyaline, septate, branched, oil granules present with KOH, hyphal end obtuse. *Clamp connections* absent in all tissues.

Habit and habitat: Solitary to gregarious, growing on soil above subterranean termite nests.

Edibility: Edible.

Additional material examined: THAILAND. Chiang Mai University, near Tat Chomphu Reservoir, 18°48′14.8″ N 98°56′51.6″ E, elevation 342.3 m, 19 August 2022, W. Phonrob (SDBR-CMUNKN1261). GenBank accession numbers PQ897229 (nrLSU) and PV020682 (mtSSU).

Notes: Morphologically similar species, namely *T. clypeatus* R. Heim, *T. striatus*, *T. aurantiacus* (R. Heim) R. Heim, and *T. tylerianus* Otieno, can be confused with *T. salmonicolor*. *Termitomyces clypeatus* differs by having a larger pileus (25–140 mm in diam.) with a sharp spiny perforatorium (5–12 mm high), a long cylindrical stipe, longer pseudorhiza (up to 220 mm long), larger basidiospores (6.0–9.0 × 4.0–6.0 µm), and narrow suprapellis hyphae (2.0–5.0 µm) [52]. *Termitomyces striatus* has a white to pale grey or greyish brown pileus, larger basidiospores (5.5–8.0 × 3.5–5.5 µm), and narrow pileipellis hyphae [52]. *Termitomyces aurantiacus* has a larger pileus size and very long pseudorhiza (up to 340 mm), lacks pleurocystidia, and has narrow pileipellis hyphae (1.5–4.5 µm) [52]. *Termitomyces tylerianus*, originally described from Kenya, differs from the present species due to characteristics such as a small pileus (10–20 mm in diam.) with a sharp pointed perforatorium, no cheilocystidia and pleurocystidia, and narrow pileipellis hyphae (2.0–5.0 µm) [52]. Phylogenetically, *T. salmonicolor* is close to *T. bulborhizus*, *T. gilvus*, and *T. planiperforatorius*. *Termitomyces bulborhizus* has a larger basidiomata size, prominent bulbous base, comparatively larger basidiospores (6–9 × 4–6 µm), cheilocystidia (19–60 × 12–34 µm), and pleurocystidia (19–78 × 10–32 µm) [53]. *Termitomyces gilvus* has a larger pileus (80–130 mm in diam.), thick pileus context (40 mm), prominent bulbous base, comparatively larger basidiospores (6–8.5 × 3.7–5.4 µm), and clamp connections [21]. *Termitomyces planiperforatorius*, also collected from the same region and also lacking a bulbous base, differs due to characteristics such as a larger basidiomata with a prominent squamulose pileus surface and larger hymenial cystidia (this study).

## 4. Conclusions

*Termitomyces* species are globally recognised as a tropical delicacy, with almost all species being edible and nutritious. In this study, five new *Termitomyces* species (*T. griseobulbus*, *T. griseobrunneus*, *T. planiperforatorius*, *T. pseudoheimii*, and *T. salmonicolor*) and one species (*T. acriumbonatus*) previously identified outside of Thailand were discovered in northern Thailand and described based on a combination of morphological characteristics and multi-gene phylogenetic analyses. These newly discovered macrofungi can be used for further research in the food, pharmaceutical, and other industries. Furthermore, this finding has increased the number of *Termitomyces* species found in Thailand. The results of this study are an important step in stimulating further research on wild edible mushrooms in Thailand and may help researchers to better understand the distribution of *Termitomyces* in Asia and around the world. Future research on *Termitomyces* in Thailand should include the identification of the associated termite hosts and should consider the potential impacts of climate change, as both factors may influence species diversity, distribution, and habitat dynamics.

## Figures and Tables

**Table 1:** Taxa, voucher numbers, countries, GenBank accession numbers, references, and type sequences used in the phylogenetic analyses conducted in this study.

Taxa	Voucher Number	Country	GenBank Accession Number			Reference
			nrITS	nrLSU	mtSSU	
*Termitomyces acriumbonatus* ^T^	LAH35345	Pakistan	MT179688	MT179689	NA	[17]
*T. acriumbonatus*	LAH36362	Pakistan	MT179687	MT179690	NA	[17]
***T. acriumbonatus***	**CMUB40061**	**Thailand**	**PQ896627**	**PQ896626**	**PV020670**	**This study**
*T. assamicus* ^T^	GUBH20038	India	OQ346313	NA	NA	[23]
*T. assamicus*	GUBH20039	India	OQ976999	NA	NA	[23]
*T. aurantiacus*	DM 152E	Cameroon	NA	KY809234	KY809186	[41]
*T. aurantiacus*	tgf 82	Tanzania	NA	AY127804	AY127852	[13]
*T. brunneopileatus* ^T^	DM392	Cameroon	NA	KY809273	KY809225	[34]
*T. brunneopileatus*	DM394	Cameroon	NA	KY809244	KY809197	[34]
*T. bulborhizus*	KM128338	China	NA	KY809261	KY809213	[34]
*T. clypeatus*	KM128340	China	NA	KY809262	KY809214	[34]
*T. clypeatus*	tgf21	Malaysia	NA	AY127802	AY127850	[13]
*T. dhofarensis*	JRZ2-22-0020	Oman	OR297695	OR338598	NA	[24]
*T. dhofarensis*	RAK-22-0025	Oman	OR297696	OR338597	NA	[24]
*T. dhofarensis* ^T^	NHZ-22-001	Oman	OR297694	NA	NA	[24]
*T. entolomoides*	tgf103	Africa	NA	AY232693	AY232680	[40]
*T. eurrhizus*	tgf101	Burundi	NA	AY232694	NA	[40]
*T. floccosus* ^T^	MFLU 19–1312	Thailand		MN633305	MN701029	[15]
*T. fragilis* ^T^	HKAS 88912	China	KY214475	NA	NA	[3]
*T. fragilis*	HKAS 88909	China	KY214476	NA	NA	[3]
*T. gilvus* ^T^	BORH/FUMS-A03	Malaysia	NA	MK472701	MK478904	[21]
*T. globulus*	DM770	Cameroon	NA	KY809252	KY809204	[39]
***T. griseobulbus*** ^T^	**CMUB40062**	**Thailand**	**PQ900075**	**PQ896628**	**PV020671**	**This study**
***T. griseobulbus***	**SDBR–CMUSOU30**	**Thailand**	**PQ900076**	**PQ896629**	**PV020672**	**This study**
***T. griseobrunneus*** ^T^	**CMUB40063**	**Thailand**	**PQ899488**	**PQ896878**	**PV020673**	**This study**
***T. griseobrunneus***	**SDBR–CMUNKN1248**	**Thailand**	**PQ899489**	**PQ899490**	**PV020676**	**This study**
***T. griseobrunneus***	**SDBR–CMUWP116**	**Thailand**	**NA**	**PQ896901**	**PV020674**	**This study**
***T. griseobrunneus***	**SDBR–CMUNKN1211**	**Thailand**	**NA**	**PQ899491**	**PV020675**	**This study**
*T. heimii*	KM16528	Malaysia	NA	KY809253	KY809205	[34]
*T. heimii*	Muid.sn	Asia	NA	AF042586	AF357091	[38]
*T. infundibiliformis*	KM144302	Cameroon	NA	KY809245	NA	[34]
*T. intermedius*	HKAS 117638	China	ON557369	ON556484	ON557367	[16]
*T. intermedius*	HKAS 117639	China	ON557370	ON556485	ON557368	[16]
*T. islamabadensis* ^T^	LAH!36788	Pakistan	MW520178	OM100949	NA	[18]
*T. islamabadensis*	LAH!36789	Pakistan	MW520179	OM100950	NA	[18]
*T. le-testui*	DM150G	Cameroon	NA	KY809231	KY809184	[34]
*T. le-testui*	KM128346	China	NA	KY809263	KY809215	[34]
*T. mammiformis*	DM25E	Cameroon	NA	KY809229	KY809182	[34]
*T. mammiformis*	DM25G	Cameroon	NA	KY809230	KY809183	[34]
*T. mboudaeinus*	DM223	Cameroon	NA	KY809274	KY809226	[34]
*T. mboudaeinus*	DM223E	Cameroon	NA	KY809237	KY809189	[34]
*T. medius*	dka 138	Cameroon	NA	AY127796	AY127844	[13]
*T. medius* f. *ochraceus*	DM602B	Cameroon	NA	KY809246	KY809198	[34]
*T. microcarpus*	tgf28	Cameroon	NA	AY127799	AY127847	[13]
*T. microcarpus*	DUKE-PRU3900	NA	AF357023	AF042587	AF357092	[39]
*T. pakistanensis*	HT1	Pakistan	OP688120	NA	NA	[20]
*T. pakistanensis*	AZ1	Pakistan	OP688121	NA	NA	[20]
***T. planiperforatorius*** ^T^	**CMUB40064**	**Thailand**	**NA**	**PQ896983**	**PV020677**	**This study**
***T. planiperforatorius***	**SDBR–CMUNKM1852**	**Thailand**	**NA**	**PQ896985**	**PV020678**	**This study**
***T. pseudoheimii*** ^T^	**CMUB40069**	**Thailand**	**PQ897224**	**PQ897223**	**PV020679**	**This study**
***T. pseudoheimii***	**SDBR–CMUNKP2013**	**Thailand**	**PQ897225**	**PQ897226**	**PV020680**	**This study**
*T. radicatus*	MRNo173	Thailand	LC068787	NA	NA	UnP
*T. robustus*	KM142418	Tanzania	NA	KY809265	KY809217	[34]
*T. robustus*	DM436	Cameroon	NA	KY809271	KY809223	[34]
*T. sagittiformis*	KM109566	South Africa	NA	KY809260	KY809212	[34]
***T. salmonicolor*** ^T^	**CMUB40070**	**Thailand**	**NA**	**PQ897227**	**PV020681**	**This study**
***T. salmonicolor***	**SDBR–CMUNKN1261**	**Thailand**	**NA**	**PQ897229**	**PV020682**	**This study**
*T. schimperi*	DM24E	Cameroon	NA	KY809228	KY809181	[34]
*T. schimperi*	tgf18	Zimbabwe	NA	AY232712	AY232686	[40]
*T. sheikhupurensis* ^T^	SKP124	Pakistan	MT192217	MT192228	NA	[19]
*T. sheikhupurensis*	SKP224	Pakistan	MT192218	NA	NA	[19]
*T. singidensis*	tgf74	Tanzania	NA	AY232713	AY232687	[40]
*T. srilankensis* ^T^	FUOR0016AGS	Sri Lanka	ON685313	NA	NA	[22]
*T. striatus*	BR5020169404421	Congo	OP179299	OP168080	OP179293	[16]
*T. striatus*	BR5020168468769	Rwanda	OP179297	OP168081	OP179294	[16]
*T. striatus* f. *bibasidiatus*	DM280B	Cameroon	NA	KY809241	KY809193	[34]
*T. striatus* f. *subclypeatus*	DM370B	Cameroon	NA	KY809268	KY809220	[34]
*T. subumkowaan*	DM260F	Cameroon	NA	KY809239	KY809190	[34]
*T. subumkowaan* ^T^	DM260B	Cameroon	NA	KY809275	KY809227	[34]
*T. tigrinus*	HKAS 107560	China	MT683156	MT679729	MT683152	[16]
*T. tigrinus* ^T^	HKAS 107561	China	MT683157	MT679730	MT683153	[16]
*T. titanicus*	tgf94	Burundi	NA	AY127801	AY127849	[13]
*T. titanicus*	KM142416	Zambia	NA	KY809264	KY809216	[34]
*T. upsilocystidiatus* ^T^	MFLU 19–1289	China	NA	MN636637	MN636642	[15]
*T. yunnanensis* ^T^	HKAS 124501	China	OP179295	OP168083	OP179290	[16]
*T. yunnanensis*	HKAS 124502	China	OP179296	OP168084	OP179291	[16]
*Termitomyces flavus* ^T^	YAAS 2021081127	Thailand	PP264695	PP264703	PP264701	[29]
*Termitomyces flavus*	YAAS 2021081126	Thailand	PP264696	PP264704	PP264702	[29]
*Asterophora lycoperdoides*	CBS170.86	NA	AF357037	AF223190	AF357109	[42]
*A. parasitica*	CBS683.82	NA	AF357038	AF223191	AF357110	[42]
*Lyophyllum shimeji*	Lc42	NA	AF357060	AF357078	AF357137	[42]
*L. decastes*	JM87/16	NA	AF357059	AF042583	AF357136	[42]

“NA” = indicates that no data are available in the GenBank database or could not be obtained. “UnP” = Unpublished; “T” = Holotype specimen.

## Data Availability

The DNA sequence data obtained from this study were deposited in GenBank (https://www.ncbi.nlm.nih.gov/nucleotide, accessed on 18 April 2025) under the following accession numbers: nrLSU (PQ896626, PQ896628, PQ896629, PQ896878, PQ896983, PQ896985, PQ896901, PQ897223, PQ897226, PQ897227, PQ897229, PQ899490, and PQ899491), nrITS (PQ896627, PQ897224, PQ897225, PQ899488, PQ899489, PQ900075, and PQ900076), and mtSSU (PV202670 to PV202682). Specimens of the new taxa identified in this study have been deposited in MycoBank (https://www.mycobank.org/, accessed on 20 April 2025) under numbers MB857473 to MB857477. The alignment of the concatenated nrLSU, mtSSU, and nrITS sequences was deposited in Zenodo under the DOI number 10.5281/zenodo.15528282.
